# Simple questionnaires outperform behavioral tasks to measure socio-emotional skills in students

**DOI:** 10.1038/s41598-021-04046-5

**Published:** 2022-01-10

**Authors:** Mélusine Boon-Falleur, Adrien Bouguen, Axelle Charpentier, Yann Algan, Élise Huillery, Coralie Chevallier

**Affiliations:** 1 Institut Jean Nicod, Département d’études Cognitives, Ecole Normale Supérieure, EHESS, Université PSL, 75005 Paris, France; 2 Santa Clara University, Santa Clara, CA 95053 USA; 3 DEPP, MENJS, 75015 Paris, France; 4 HEC Paris, Paris, France; 5 LEDa, Université Paris-Dauphine, Université PSL, IRD, CNRS, 75016 Paris, France; 6 LNC², Département d’études cognitives, Ecole normale supérieure, Université PSL, INSERM, 75005 Paris, France

**Keywords:** Human behaviour, Personality

## Abstract

Recent empirical research has shown that improving socio-emotional skills such as grit, conscientiousness and self-control leads to higher academic achievement and better life outcomes. However, both theoretical and empirical works have raised concerns about the reliability of the different methods used to measure socio-emotional skills. We compared the reliability and validity of the three leading measurements methods—a student-reported questionnaire, a teacher-reported questionnaire, and a behavioral task—in a sample of 3997 French students. Before analyzing the data, we polled 114 international researchers in cognitive development and education economics; most researchers in both fields predicted that the behavioral task would be the best method. We found instead that the teacher questionnaire was more predictive of students’ behavioral outcomes and of their grade progression, while the behavioral task was the least predictive. This work suggests that researchers may not be using optimal tools to measure socio-emotional skills in children.

## The importance of socio-emotional skills

What makes a student successful in school? Studies have shown that socio-emotional skills such as self-control or self-esteem rival cognitive skills in predicting academic achievement^1–10^ and other life outcomes such as employment, earnings, health, and criminality^11–14^. Among these socio-emotional skills, conscientiousness, self-control, and grit have been identified as playing an important role for academic achievement and future life outcomes^15–20^. Recent empirical works have recently demonstrated that interventions to increase these skills in children led to an increase in academic performance, suggesting a causal link between these variables^21–25^. In light of these findings, policy makers and practitioners around the world have implemented programs aimed at developing socio-emotional skills in students (e.g., CASEL or KIPP charter schools in the United States, the Singapour Positive Education Network, the Contruye-T program in Mexico, the Beyond Academic Learning Program of the OECD, and Energie Jeunes in France).

The domain of socio-emotional skills is the subject of interdisciplinary research, spanning fields from economics to development psychology and professionals from academics to educators. As a result, many different terms and theoretical frameworks are used to describe and understand these skills. For example, in the economics literature, scholars often use the phrase “non-cognitive skills”, while some education experts prefer to talk about “character skills”. In this article, we use the term socio-emotional skills as it is widely used in the psychological literature, and define such skills in accordance to the OECD framework as individual capacities that can be manifested in consistent patterns of thoughts, feelings and behaviors^26^.

## Measurement issues with socio-emotional skills

Imprecise measures of socio-emotional skills will lead to imprecise conclusions and possibly misleading policy recommendations, especially in small sample studies^27^. Therefore, researchers and policy makers should be cautious when selecting tools to measure socio-emotional skills. Measures of skills must be both reliable, meaning that they provide similar results when repeated under the same conditions, and valid, meaning that they are sufficiently correlated to the underlying construct. Three main methods are currently used in the literature –self-reported questionnaires, third-party questionnaires (e.g., parents or teachers), and behavioral tasks– each of which is exposed to potential biases^28^. Self-reported questionnaires are affected by the “social desirability bias”, which arises when respondents consciously or unconsciously provide answers that might be viewed favorably by others. They can also be affected by the fact that different people can have different standards or reference points, which may lead two people that are objectively identical on a given trait (say self-control or grit) to report different scores on that trait because the reference point of their group is different. Similarly, measures can include contingencies both in terms of items within them and how measures are utilized, such as asking whether a student pays attention “never”, “sometimes”, “often”, or “almost always”. In such cases, people’s responses may be influenced by the context (for example paying attention in class versus paying attention at what parents are saying) or the reference point for an answer (what “often” might mean to different people). In addition, responses may be affected by the cognitive skills of the respondent and her ability to understand the questions asked^29,30^. Third-party questionnaires are also exposed to the “reference bias” and may be further flawed by subjective impressions or by misinterpretations of behavior, as well as poor observation and thus poor information on that behavior. By contrast, behavioral tasks provide objective measures and are less affected by these biases. However, they are influenced by cognitive factors such as response time, accuracy or IQ which can be unrelated to the construct at hand^31^. In addition, behavioral tasks measure performance at the time of the experiment, and not the average level of performance over a longer period of time^32^. For a detailed account of potential limitation with each method of measure, see^33^ and^34^.

## The present research

Despite these validity concerns, no study has directly compared different methods to measure socio-emotional skills. The overall purpose of this paper is to compare the reliability of student-reported questionnaires, teacher-reported questionnaires, and a behavioral task to measure socio-emotional skills and to identify which is the most valid tool. To shed light on this issue, we measured conscientiousness (the desire to do a task well), self-control (the ability to regulate behavior, attention and emotions in the service of valued goals), and grit (the ability to persevere towards long-term goals) using three standardized methods of measurement. We selected methods that have been validated for their reliability and consistency and are widely used in experimental works^22,35^. The student-reported questionnaire includes items related to conscientiousness from the Big Five Inventory^36^, the Short Grit Scale^37^, and the Domain-Specific Impulsivity Scale for children^38^ which measures self-control. The teacher-reported questionnaire was composed of the Character Growth Card^39^, a tool used in many educational programs, such as the 270 KIPP schools in the United States, to measure students’ socio-emotional skills, including self-control and grit, and shown to correlate with GPA, class participation and peer conflic. For the behavioral task, we used the Academic Diligence Task, an experimentally validated tool to measure self-control and grit in students, showing convergent validity with self-ratings of Big Five conscientiousness and its facets: self-control and grit. The task also demonstrates incremental predictive validity for objectively measured GPA and other measures of academic achievement^40^. During this task, students had to chose between solving simple math problems or watching entertaining videos. Before the beginning of the task, the experimenter explained that solving math problems is important to develop the brain and students were encouraged to solve as many math problems as possible. Students were also told that their answers would be anonymous and confidential, and that they were completely free to pick either math exercises or videos. The task consisted of three block of three minutes. These three methods are used interchangeably in the literature to measure grit, self-control, and conscientiousness in students.

We collected data in a sample of 97 French REP middle schools located across the country (Réseau d’Éducation Prioritaire are schools receiving aid from the government to address the academic and socioeconomic needs of students). A total of 3997 students were randomly selected among all sixth and seventh grade’s students to complete the Academic Diligence Task and a student-reported questionnaire during normal school hours. One teacher per class completed the Character Growth Card questionnaire for each student. In addition to these measures of non-cognitive skills, we also collected data from school records. For each student, we recorded the number of late arrivals, the number of absences, the number of sanctions, and the number of disciplinary actions during the school year (sanctions are often hours of detention, while disciplinary actions are more severe than sanctions and are decided collectively by the school administration). We also recorded their math and French GPA. Students were asked how much time they had spent on their homework in the last two days. The data collection process took place over several years, allowing for a sub-sample of students to be randomly drawn in both their sixth and seventh grade. Students were 13 years old on average (SD = 0.78), 88% were of French nationality, and 52% benefited from financial aid, which is about 14 points above the national rate. On average, students arrived 4.8 times late at school during the year (SD = 8.3), has a total of 3.0 days of unjustified absences (SD = 6.8), received 3.5 sanctions (SD = 7.5) and 0.3 disciplinary actions (SD = 1.2). We standardized, inverted and summed these four measures to create a disciplinary index, higher values indicating more disciplined behavior. French and math average GPA were respectively 12.1 and 11.9 (SD = 3.4 and 3.9). From the student-reported questionnaire, we obtained a measure of conscientiousness, self-control, and grit. From the teacher questionnaire, we obtained a measure of self-control and grit. From the behavioral task, we retrieved the number of questions attempted, the number of questions correctly solved (productivity), and the percentage of time spent solving questions versus watching videos. These variables measure the ability of students to work diligently while resisting distractions, and have been shown to be correlated to grit, self-control, and conscientiousness. All the data analysis was conducted using the statistical software STATA.

To compare these measures, we first assessed their reliability, i.e. whether each measure is consistent across time, raters, and items, using long-term stability and Cronbach’s Alpha. Second, we assessed the validity of each method by testing whether the measures of socio-emotional skills correlate with behaviors related to the same psychological constructs. Time spent doing homework and discipline at school are both behavioral measures that we expect to correlate highly with conscientiousness, self-control, and grit. In addition, the literature has shown that these socio-emotional skills have an impact on the development of linguistic, cognitive, and academic aptitudes^21^. We therefore expect that students who rate higher on conscientiousness, self-control, and grit scales will be more likely to have higher grades or to see their grades improve over time. Valid measures should thus predict school behavior and academic performance.

**Figure 1:**
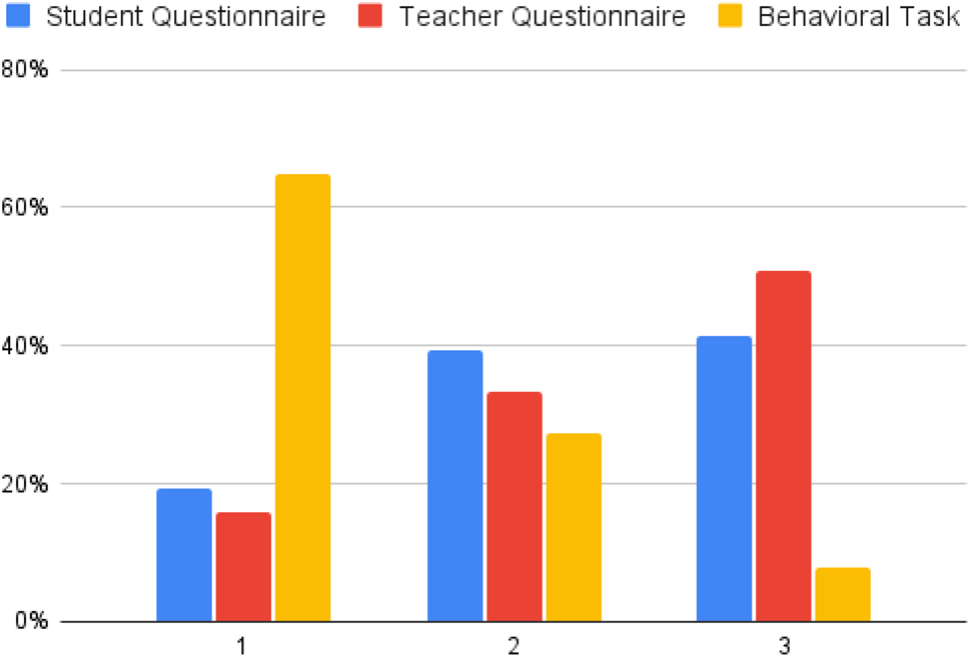
Results from researchers survey. A total of 114 researchers were surveyed online and ranked the three measures of socio-emotional skills from (1) best method to (3) worst method. Amongst these researchers, 45 came from the field of economics, 36 from the cognitive and psychology sciences, and 33 from other fields such as biology or anthropology. The table shows the percentage of researchers that selected a specific method for each rank. The majority of researchers (65%) ranked the behavioral task as the best method.

Finally, we polled a sample of 114 researchers in both the economics and cognitive sciences before completing the data analysis to prevent hindsight bias, that is, the tendency for researchers to think “I knew that already”^41^. A link was sent out to researchers via the network of the Paris School of Economics, the École Normale Supérieure, and Université Paris Dauphine. The survey asked respondents to rank the three measures of socio-emotional skills in middle-schoolers: (1) standardized child self-reported questionnaire, (2) standardized teacher-reported questionnaire, and (3) standardized behavioral task. We found that a vast majority of researchers believe that behavioral tasks are better than teacher-reported questionnaires (81%) or believe that behavioral tasks are better than self-reported questionnaires (76%). Detailed results can be seen in Fig. 1. The respondent’s research speciality—economics or cognitive science—did not affect responses.

## Results

### Reliability of the measures

Assessment of reliability showed that all three measures of socio-emotional skills were similarly reliable, with a small advantage for the teacher-reported questionnaire (Table 1). We first calculated Cronbach’s alpha and McDonald’s omega for each measure. For the questionnaires, we computed the inter-item correlation for each socio-emotional skill. For the behavioral task, we computed the correlation between each of the three blocks for each variable (number of subtractions attempted and solved, and percentage time spent on solving subtractions versus watching videos). Our results show that the teacher-reported questionnaire had the highest Cronbach alpha (0.93–0.94), while the behavioral task had the lowest alpha (0.44–0.58). Values ranging from 0.70 to 0.95 are considered high in the literature^42^. We found similar results for McDonald’s omega. Although Cronbach’s alpha and McDonald’s omega are widely used in the psychology literature to assess reliability, these measure are sensitive to many factors such as the number of items. A complementary approach to assess reliability is to assess long-term stability^43,44^. Indeed, as argued by McCrae et al. “personality traits are, by definition, enduring dispositions; measures that fail to show long-term stability cannot be valid trait measures”^43^. However, if conscientiousness, self-control, and grit are more aptitudes than traits, we should expect them to change over time. Evidence shows that conscientiousness is already stable in adolescents aged 12, however, conscientiousness tends to increase slightly for girls and decrease slightly for boys in early adolescence. To take into account such changes we also measured rank-order stability by gender and found similar results to long-term stability (see Appendix for rank-order stability)^45^. For a sub-sample of students, we have answers to the questionnaires and behavioral task in both their sixth and seventh grade. We can therefore test the correlation from one year to the next. We expected the long-term stability for the teacher questionnaire to be lowest given that we tested the correlation of answers from two different teachers, whereas the student questionnaire and the task was completed by the same student in both years. Table 1 shows that all methods of measure show similar long-term stability (ranging from 0.41 to 0.54, p = 0.01), indicating that these different methods are similarly reliable. These coefficients are similar to the ones found in the literature^44^. Although teachers talk amongst themselves and observe the same student in similar situations, it is interesting to note that teachers’ answers correlate as much from one year to the next as do the answers of students about themselves. We conclude from this evidence that if anything, the teacher-reported questionnaire is more reliable than the other two methods.

**Table 1:** Reliability of each method of measure. The table shows the $\alpha$ coefficient corresponding to Cronbach’s Alpha for each measure of socio-emotional skill, McDonald's omega, as well as the number of items includes in the coefficient. The table also shows the partial correlation coefficient from one year to the next for each measure, and the sample size for which this correlation was calculated.

	Reliability			Long-term Stability	
	$\alpha$	$\omega$	N items	Corr	*N*
**Student-reported**					
Conscientiousness	0.70	0.71	4	$$0.49^{***}$$	1,402
Self-control	0.82	0.82	8	$$0.53^{***}$$	1,417
Grit	0.65	0.65	8	$$0.48^{***}$$	1,434
**Teacher-reported**					
Self-control	0.94	0.94	8	$$0.54^{***}$$	906
Grit	0.93	0.93	3	$$0.48^{***}$$	905
**Behavioral task**					
Attempted	0.54	0.56	3	$$0.49^{***}$$	1,435
Solved	0.58	0.60	3	$$0.51^{***}$$	1,435
Time on task	0.44	0.47	3	$$0.41^{***}$$	872

### Validity of the measures

**Figure 2:**
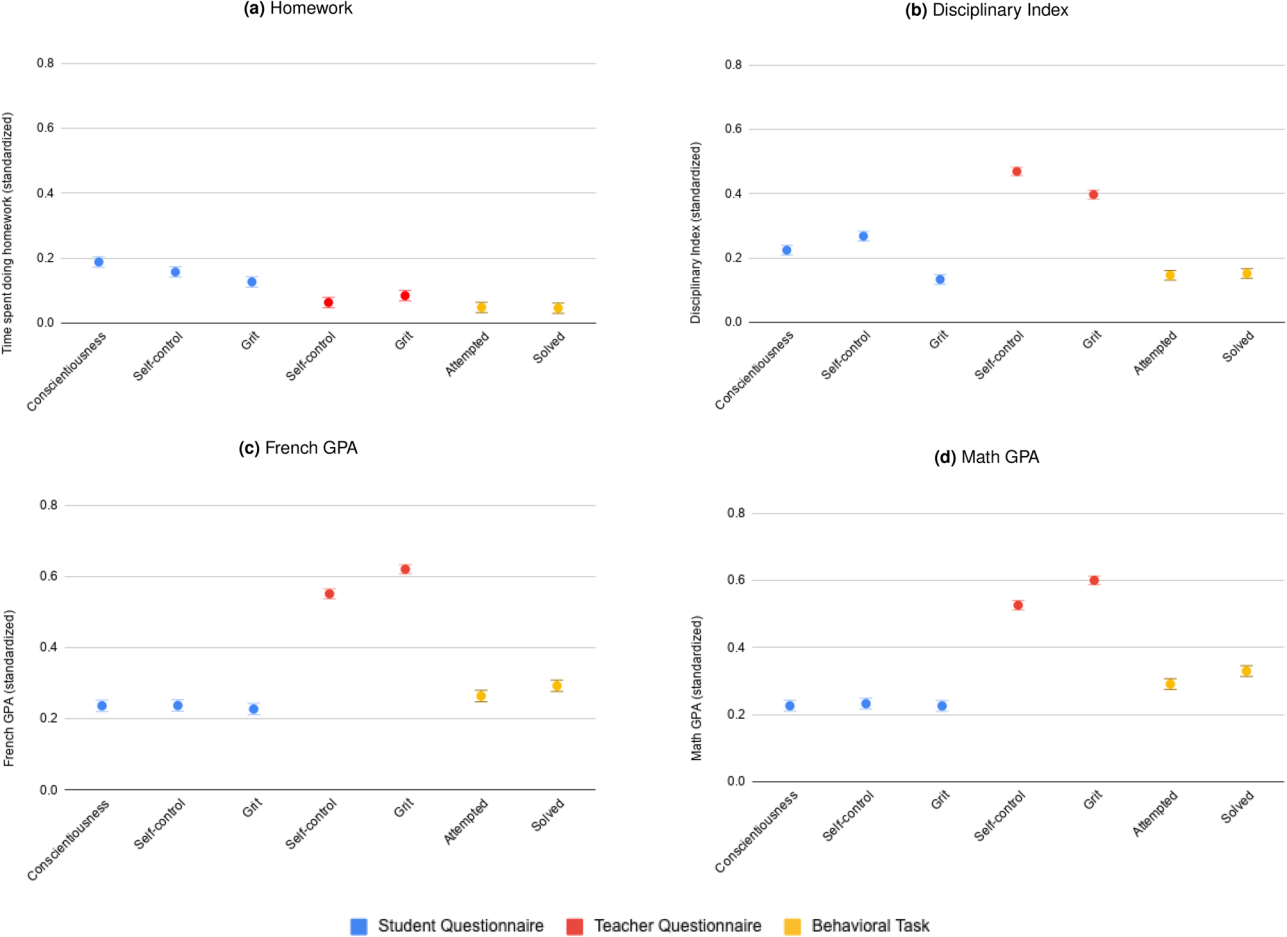
Standardized correlation coefficients of the ordinary least square regression between socio-emotional skills and students’ outcomes (N = 3,997), adjusted for school fixed effects and school year fixed effects (grade 6 and grade 7). Observations with missing outcomes or covariate data were excluded from the sample. The blue points represent the student-reported measures, the red points represent the teacher-reported measures and the yellow points represent the behavioral task measures. The error bars indicate plus or minus one standard deviation. Four outcome measures are presented: time spent doing homework (**a**), disciplinary index (**b**), French GPA (**c**) and Math GPA (**d**). Time spent doing homework (**a**) is the standardized student-reported sum of time spent doing homework in the last two days. The regression controls for the day the data was collected. Disciplinary index (**b**) is the sum of the standardized number of late arrivals, absences, sanctions and disciplinary actions. French GPA (**c**) is the standardized grade received by the student in French. Math GPA (**d**) is the standardized grade received by the student in math. The effect is statistically different from 0 for each variable.

Contrary to our own expectation and to researchers’ predictions, our results show that the behavioral task is the least valid method to assess socio-emotional skills in students. We first tested the correlation between each measure and the time spent doing homework. Results in Fig. 2a show that the student questionnaire was most correlated with time spend doing homework (0.13–0.19, p = 0.01) compared to the teacher questionnaire (0.06–0.08, p = 0.01) and the behavioral task (0.05, p = 0.05). However, the $R^2$ for these regressions is quite low ($R^2$ ranging between 0.03 and 0.06). Given that time spent doing homework was a self-reported variable, we can suspect measurement errors due to social desirability bias. Moreover, students prone to social desirability bias may over-declare both time spent on homework and their socio-emotional skills, which may explain the higher correlation between the two. Therefore, we also look at an objective measure of behavior: the discipline index of students, which is based on school administrative records of absenteeism, tardiness, sanctions and disciplinary actions. We find that, as expected, all socio-emotional measures are positively correlated to the disciplinary index, meaning that students who are more conscientious, gritty, or have higher self-control are more disciplined. Results in Fig. 2b also show that the teacher-reported questionnaire is more strongly correlated with the discipline index (0.40–0.47, p = 0.01) than the student questionnaire (0.13–0.27, p = 0.01) or the behavioral task (0.15, p= 0.01) are. We perform the same analysis for French and math GPA in panel 2c and 2d and similarly we find that the teacher questionnaire is most predictive.

**Figure 3:**
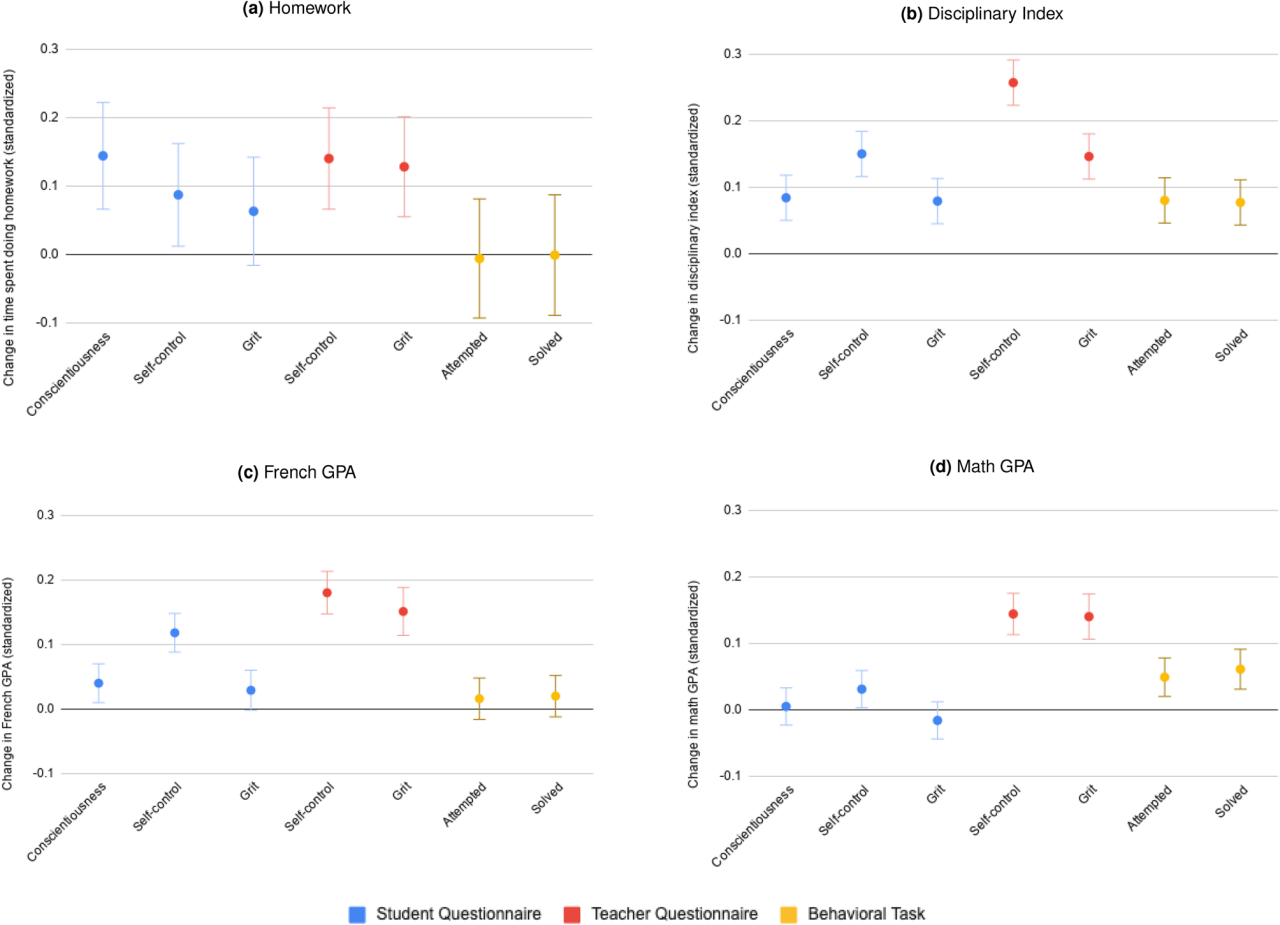
Standardized correlation coefficients of the ordinary least square regression between socio-emotional skills and student outcomes, adjusted for school fixed effects and school year fixed effect (grade 6 and grade 7). Observations with missing outcomes or covariate data were excluded from the sample. The blue points represent the student-reported measures, the red points represent the teacher-reported measures and the yellow points represent the behavioral task measures. The error bars indicate plus or minus one standard deviation. Four outcome measures are presented: change in time spent doing homework (**a**), change in disciplinary index (**b**), change in French GPA (**c**) and change in math GPA (**d**). Change in time spent doing homework (**a**) is the difference between the standardized student-reported sum of time spent doing homework in the last two days in sixth and seventh grade, N = 191. The regression controls for the day the data was collected. Change in disciplinary index (**b**) is the difference between the sum of the standardized number of late arrivals, absences, sanctions and disciplinary actions in sixth and seventh grade, N = 559. Change in French GPA (**c**) is the difference between the standardized grade received by the student in French in sixth and seventh grade, N = 491. Change in math GPA (**d**) is the difference between the standardized grade received by the student in math in sixth and seventh grade, N = 527.

One possible interpretation for the high correlation between the teacher reported questionnaire and observed behavior is that teachers use information on grades, absences, tardiness, sanctions and disciplinary actions when filling the Character Growth Card. Under this interpretation, teachers would not be any better at evaluating socio-emotional skills, they would simply have better access to objective behavioral outcomes. In order to rule out this interpretation, we correlate the teacher-reported questionnaire with behavioral outcomes that the teacher cannot directly observe. Specifically, we check if the teacher-reported questionnaire in a given year correlates with the student’s disciplinary index and grades during the following year, controlling for the student’s current year outcome. In other words, we test whether each measure of socio-emotional skills predicts *progress* in behavioral outcomes from one year to the next. The theory predicts that students who are more conscientious, gritty, or have more self-control should see higher improvements in their discipline index and GPA than students who are less conscientious, gritty, and are more impulsive. We find that, controlling for current current behavioral outcome, the teacher-reported questionnaire consistently predicts behavioral outcomes in the following year (see Fig. 3). Once again, teacher-reported questionnaire perform significantly better than student-reported questionnaires or behavioral tasks at predicting future behavior. Importantly, the teacher-reported questionnaire is the only socio-emotional measure that significantly and consistently predicts future academic results, as shown in Fig. 3c,d. We also test whether behavioral measures in grade 6 predict a change in behavior or grades from grade 7 to grade 8 and from grade 8 to grade 9, and whether behavioral measures in grade 7 predict a change in behavior or grades from grade 8 to grade 9 and find similar results (See Appendix Figures C16 to C19). Taken together, these results show that the teacher-reported questionnaire is a better predictor of future behavioral outcomes that are intimately related to the construct of interest.

### Comparing the costs of different methods

For a method of measure to be useful, it must not only be reliable and valid, but it must also be implementable in practice^34^. For this reason, we were interested in the relative cost of each method of measure. Our analysis included the cost of hiring research assistants, questionnaire, and task support (electronic or paper), data transcription, data cleaning, etc. We estimated the cost of each method to be 44,731 euros for the student questionnaire, 12,907 euros for the teacher questionnaire, and 50,191 euros for the behavioral task (see Table 2 for a more detailed breakdown of costs). Although specific costs may vary significantly from one study to the next, a number of factors make behavioral tasks consistently costlier than questionnaires. In addition, one teacher can be surveyed for multiple students, which creates economies of scale. Another way to look at cost is the money spent per student to predict a change of one standard deviation in behavior. Based on their respective predictive power of change in disciplinary index, we find that the teacher questionnaire is only 13 euros per student, while the student questionnaire and the behavioral task are respectively 75 euros and 154 euros per student. Our study suggests that many research teams might be allocating resources to the implementation of behavioral tasks, although cheaper and more accurate methods of measure exist.

**Table 2:** Breakdown of experimental costs. This table shows the breakdown of costs for each method of measure. Information includes costs pertaining to phoning schools and printing information for consent. Support includes the cost of paper and mailing for teacher questionnaires and digital tablets for student questionnaires and for the behavioral task. The total cost per sd per student is the cost that is needed to observe one standard deviation in the change of behavioral index for one student.

	Student	Teacher	Behavioral
	Questionnaire	Questionnaire	Task
**Cost breakdown**			
Information	2,125	883	2,125
Support	14,130	1,669	14,130
Software	540		6,000
Terrain	27,936		27,936
Reminder		1,746	
Double-entry		7,980	
Cleaning		630	
**Total**			
Total cost in euros	44,731	12,907	50,191
Per sd per			
Student in euros	75	13	154

## Discussion

Reliability and validity measures show that the behavioral task systematically under-performed relative to questionnaires to predict outcomes related to socio-emotional skills. In addition, the teacher-reported questionnaire, which was considered the worse method of measurement by researchers, showed similar reliability and the highest correlation with behavioral and school outcomes. It is however important to acknowledge limitations coming from our sample. The students who participated in our study are not representative of the entire French population as they attend low income schools. It may be the case that measures from a behavioral task are more reliable and valid for students with affluent backgrounds. It may also be the case that teachers in more affluent schools are less able to assess the socio-emotional skills of their students.

These results contradict researchers’ predictions and contribute to a growing literature demonstrating the limits of behavioral tasks. For example, a recent study on self-control shows that self-reported measures and inhibition task performance correlate very poorly^46^. The authors list three main reasons for the lack of convergence between self-reported measures and behavioral tasks: (1) self-reported questionnaires measure typical performance while tasks measure maximum performance, (2) self-reported measures capture central tendencies of behavior, while behavioral tasks are momentary captures of one time performance, and (3) self-reported questionnaires measure a general cross-domain trait, while a behavioral task focuses on a more narrow manifestation of the trait. A recent study by Enkavi et al. shows that self-regulation measures derived from self-reported questionnaires have higher test-retest reliability than those derived from behavioral tasks^44^. A number of studies also show that self-reported measures and behavioral tasks correlate poorly, with self-reported measures correlating better with real-life outcomes^47,48^. Finally, behavioral tasks may also suffer from framing effects. For example a study on cooperation shows that performance in a cooperation game is strongly affected by the name given to the game (Community Game, Wall Street Game, Environment Game or simply Game)^49^.

Our findings are relevant not only to the study of personality, but also to many other fields measuring individual outcomes. Studies focusing on health outcomes, for instance, have also shown that self-reported measures often predict actual morbidity, mortality or other risk factors better than supposedly more objective measures such as the Global Activity Limitation Index^50–52^. Although health institutions such as the National Health Institute (USA) have pushed for standardized ways of measuring the health of patients, these might in fact be less accurate than self-reports to predict morbidity and mortality. Including self-reported and third party questionnaires therefore remains valuable, especially in domains where experimental tasks or objective measures may not capture inter-individual differences properly. Studies of happiness and wellness could include third party questionnaires and compare the results with self-reported measures, as behavioral tasks and objective measures are hard to develop in this domain.

Does this mean we should stop using behavioral tasks altogether and rely only on self-reported or third-party questionnaires to measure socio-emotional skills? There are no perfect tools of measurement and selecting the appropriate one depends on the context of the experiment. For example, in the context of policy evaluation, self-reported questionnaires may not be reliable as the intervention may affect both behavior and the perception of the behavior. For example, a study by Algan, Guyon, and Huillery shows that following an intervention to curb school bullying, pupil-reported violence *increased* but objective and teacher-reported violence *decreased*^53^. Based on the self-report only, researchers would have falsely believed that the intervention had a negative impact, when the results are in fact due to a better awareness of bullying among pupils. A teacher-reported questionnaire may be a poor predictor of behavior if the teacher has limited contact with the student or has an incentive to bias responses (such as being compensated based on the progression of students). Behavioral tasks can be done repeatedly to obtain a more accurate measure of the average level of the trait being measured, several behavioral tasks can be combined to have a broader understanding of the construct and tasks can be improved to better capture the trait of interest. The most surprising finding of our study was the high validity of the teacher-reported questionnaire, even after controlling for observed behavior. Third-party informants is an interesting yet underused method to study psychological traits. A study by Vazire shows that informants are a cheap way to gather data, that they are often willing to cooperate and that they provide valid data^54^. Yet, one limitation of our study is that attrition is higher for the teacher-reported questionnaire than it is for the student-reported questionnaire, because it is harder to collect data from staff than from the children themselves. New approaches should be developed to ensure a high rate of teacher response. Instead of abandoning the use of questionnaires, we should work to improve them, for example by providing a shared reference point, or by ensuring that respondents believe in the confidentiality of their answer, which would minimise the influence of the social desirability bias.

Psychologists and economists believe that behavioral tasks are the most reliable way to measure socio-emotional skills. Our study allowed to test this intuition by comparing three tools to measure socio-emotional skills: a student-reported questionnaire, a teacher-reported questionnaire and a behavioral task. In addition to comparing the reliability of each tool, access to long-term behavioral data allows to compare their construct validity. We found that contrary to researchers’ predictions, the behavioral task was the least valid tool while the teacher-reported questionnaire was the most valid. Research on socio-emotional skills may suffer from a bias regarding which tools are best to use.

## Method

The experimental protocol was approved by the Ethical Research Committee of the JPAL (Abdul Latif Jameel Poverty Action Lab) in Paris. All methods were carried out in accordance with relevant guidelines and regulations.

### Participants

We collected data in a sample of 97 French REP middle schools located across the country. Written informed consent was obtained from all subjects or, for subjects under 18, from a parent and/or legal guardian. Seven students per class were randomly selected among all sixth and seventh grade’s students to participate in our study. Students were 13 years old on average (SD = 0.78), 88% were of French nationality, and 52% benefited from financial aid, which is about 14 points above the national rate. Data collection was embedded in a larger study aimed at measuring the impact of a low intensity intervention in middle school students to improve academic achievement^25^. To ensure no confounding with the larger study intervention, we sampled students only from the control group.

All students in the experiment completed the questionnaire and the behavioral task, therefore missing values came from random issues with extractions from school records. We excluded a total of 2,006 students from our sample for whom data was missing. However, to ensure that our results were not affected by attrition, we conducted the same analysis on the total sample using imputations and found no difference (see Appendix Figures C8 to C11). The data collection took place over three years. The first cohort consisted of 784 students in the sixth grade during the spring of 2015. The second cohort consisted of 1,166 students in sixth grade and 1,117 students in seventh grade in the spring of 2016. The third cohort consisted of 930 students in seventh grade in the spring of 2017. A total of 3,997 separate measures were included in our study. Among those, a subset of these measures come from the same students who were randomly selected in both the first and second cohort, or in both the second and the third cohort.

### Experimental design

Research assistants were dispatched across the French territory to collect data in each middle school. After training, research assistants collected administrative data from the school and administered both the behavioral task and the student questionnaire during normal school hours. Students each received earbuds and a digital tablet to complete both the questionnaire and the behavioral task. Research assistants also distributed the teacher questionnaire in paper format to one teacher per class and collected the answers a few days later. Research assistants and the teachers involved in the study were blind to the purpose of the experiment and the hypothesis being tested.

### Measures of socio-emotional skills

To measure socio-emotional skills, we used three separate instruments: a student-reported questionnaire, a teacher-reported questionnaire and a behavioral task. When the instruments were only available in English, the material was translated from English to French using the Back Translation method to ensure a high degree of reliability. The French version of the questionnaires can be found in the Appendix (see Materials Section in Appendix).

### Student-reported questionnaire

Students completed a battery of self-reported questionnaires on digital tablets. They were told that their answers would remain anonymous and confidential. All responses were encoded on a scale from 1: *not at all like me* to 5: *very much like me*. Some items in each scale were inverted on the questionnaire to make sure that students were not systematically choosing the same answer. Answers were re-coded and averaged such that a higher score always indicates more agreement with the construct. Students answered four questions from the Big Five Inventory to assess conscientiousness^36^. Students answered eight questions related to grit adapted from the Short Grit Scale, four questions related to consistency of interest and four questions related to perseverance of effort^37^. A grit composite index was calculated as the mean of the answer to all eight questions, with higher scores indicating more grit. Students answered eight questions related to self-control from the Domain-Specific Impulsivity Scale for children^38^. Four questions were related to self-control in the domain of school work and interpersonal relationships. A self-control composite index was calculated as the mean of the answer to all eight questions, with higher scores indicating more self-control.

### Teacher-reported questionnaire

One teacher per class completed a questionnaire during normal school hours. Questionnaires were translated from the Character Growth Card^39^ and the answers ranged from 1: *this doesn’t resemble the student at all* to 5: *this completely resembles the student*. Within the Character Growth Card, teachers had to answer three questions related to grit and eight questions related to self-control for each student. A grit composite index was calculated as the mean of the answer to all three questions, with higher scores indicating more grit. A self-control composite index was calculated as the mean of the answer to all eight questions, with higher scores indicating more self-control.

### Behavioral task

For the behavioral task, we replicated the Academic Diligence Task developed in Galla et al., 2014; the pre-registration for the replication is available on the project’s OSF page https://osf.io/afzgx^40^. This task was designed to measure self-control and grit in students. Students had to choose between solving simple math questions and watching entertaining videos (e.g., a movie trailer or music videos). Before the beginning of the task, the experimenter explained that solving math problems is important to develop the brain and students were encouraged to solve as many math problems as possible. Students were also told that their answers would be anonymous and confidential, and that they could do whatever they wanted. The task consisted of three blocks of three minutes during which the students could choose between solving one digit subtractions or watching entertaining videos. Our outcome variables were the total number of attempted subtractions, the total number of subtractions correctly solved, and the percentage of time they spent solving math problems. For a complete description of the task and replication, please see the Appendix.

### Measures of behavioral outcomes and academic achievement

For each student, we extracted data on disciplinary outcomes from school records: number of late arrivals, number of absences, number of sanctions, and number of disciplinary actions. On average, students arrived 4.8 times late at school during the year (SD = 8.3), has a total of 3.0 days of unjustified absences (SD = 6.8), received 3.5 sanctions (SD = 7.5) and 0.3 disciplinary actions (SD = 1.2). We standardized, inverted and summed these four measures to create a disciplinary index, higher values indicating more disciplined behavior. Each student was also asked to report the time she spent on homework in the last two days in the student-reported questionnaire. Given that time spent doing homework may be sensitive to the day of the week, we recorded the day of the week when the student completed the survey and added it as a control. In addition, we also collected students’ math and French GPA. French and math average GPA were respectively 12.1 and 11.9 out of 20 (SD = 3.4 and 3.9). For a subset of students, we had access to their school records in the years following our study. This allowed us to measure the change in the disciplinary index and the change in French and math GPA from one year to the next for these students.

### Statistical analysis

To estimate the validity of each method of measure, we used ordinary least square to estimate the following equation:

$$\begin{aligned} Y_{is} = \alpha + \beta NC_{is} + \gamma X_{is} + \theta _{s}+ \epsilon _{is} \end{aligned}$$

where $Y_{is}$ is the standardized behavioral or school outcomes (e.g., time spent doing homework, disciplinary index, French or math GPA) for individual *i* in school *s*, $NC_{is}$ is the standardized measure of a socio-emotional skill (e.g., BIG5 conscientiousness score, number of subtractions correctly solved, etc.), $X_{is}$ is a vector of baseline covariates (including the school year of the student), $\theta _{s}$ are school fixed effects, and $\epsilon _{is}$ is an error term. In order to control for current behavioral outcomes, we estimated a similar equation:

$$\begin{aligned} Y_{is, t+1} = \alpha + \delta Y_{is, t} + \beta NC_{is, t} + \gamma X_{is, t} + \theta _{s}+ \epsilon _{is} \end{aligned}$$

where $Y_{is, t+1}$ is the behavioral outcome (e.g., time spent doing homework, disciplinary index, French or math GPA) in the school year $t+1$ and $Y_{is, t}$ is the behavioral outcome the school year *t* and $NC_{is, t}$ is the standardized measure of socio-emotional skills in school year *t*. To test for potential effect of mitigating factors, we ran the same regressions adding age, gender, nationality (french or foreign) and financial aid (yes or no) as dummies. We find that all effects were robust to adding these covariates.

## Supplementary Information

Supplementary Information.

## Data Availability

The datasets and codes generated during and/or analysed during the current study are available in the Open Science Framework repository, https://osf.io/afzgx/.
