# Supplementary material for: Simple questionnaires outperform behavioral tasks to measure socio-emotional skills in students
Source: Sci Rep. 2022 Jan 10;12:442. doi: 10.1038/s41598-021-04046-5 (PMC8748826; doi:10.1038/s41598-021-04046-5)
Supplement: Supplementary file 1 — Supplementary Information. [file 41598_2021_4046_MOESM1_ESM.docx]

SUPPLEMENTARY MATERIAL: Simple questionnaires outperform behavioral tasks to measure socio-emotional skills in students

Mélusine Boon-Falleur^a,1^, Adrien Bouguen^b^, Axelle Charpentier^c^, Yann Algan^d^, Élise Huillery^e, 2^, and Coralie Chevallier^f, 2^

^a^Institut Jean Nicod, Département d’études cognitives, ENS, EHESS, PSL University, 29 rue d’Ulm, 75005 Paris, France

^b^Santa Clara University, 500 El Camino Real, Santa Clara, CA 95053, USA

^c^DEPP, Ministry of Education, 65 rue Dutot, 75015 Paris, France

^d^Sciences Po, Observatoire Français des Conjonctures Economiques, Paris, France, 28, rue des Saints-Pères, 75006 Paris, France

^e^Université Paris Dauphine, PSL, Place du Maréchal de Lattre de Tassigny, 75016 Paris, France

^f^Laboratoire de Neurosciences Cognitives et Computationnelles, INSERM, ENS, PSL University, 29 rue d’Ulm, 75005 Paris, France

^1^Corresponding authors: mboonfalleur@clipper.ens.fr; coralie.chevallier@ens.fr

^2^contributed equally

Appendix A

Materials

When the materials (student questionnaire, Academic Diligence Task, etc.) were only available in English, the material was translated from English to French using the Back Translation method to ensure a high degree of reliability.

***Figure A1.*** Expert survey design. 114 experts completed this survey online before we analyzed data to evaluate each method of measurement. Experts came from the network of the Ecole Normale Supérieure, Université Paris Dauphine and the Paris School of Economics.


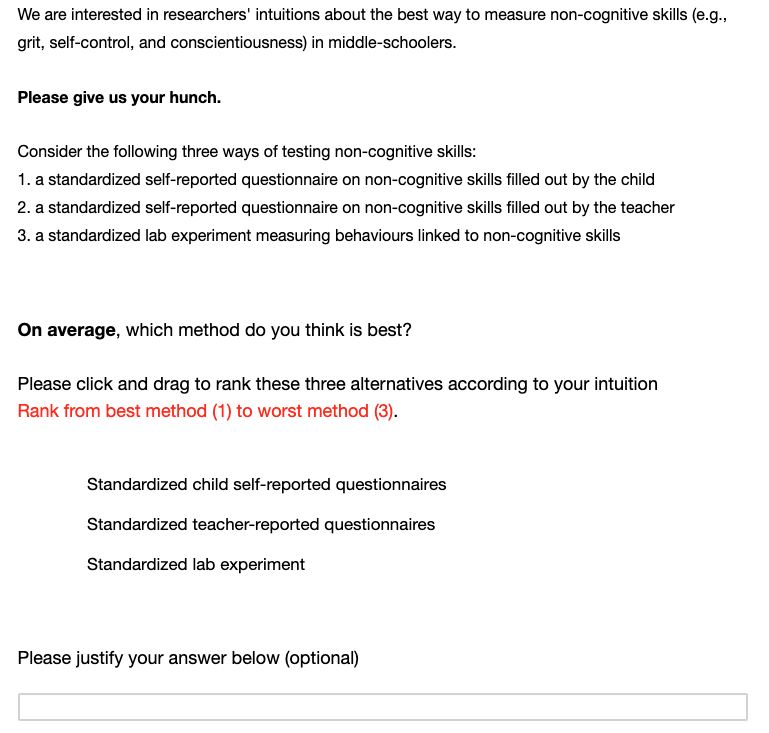


***Figure A2.*** French version of the Character Growth Card. This constituted the teacher questionnaire to evaluate the socio-emotional skills of students. The English version of the character growth card can be found in Park et al. (2017) Park, Tsukayama, Goodwin,Patrick, and Duckworth (2017). Teachers who completed the survey for students were the “professeur principal” for the class, meaning that they are responsible for a group of 30 students throughout the academic year.


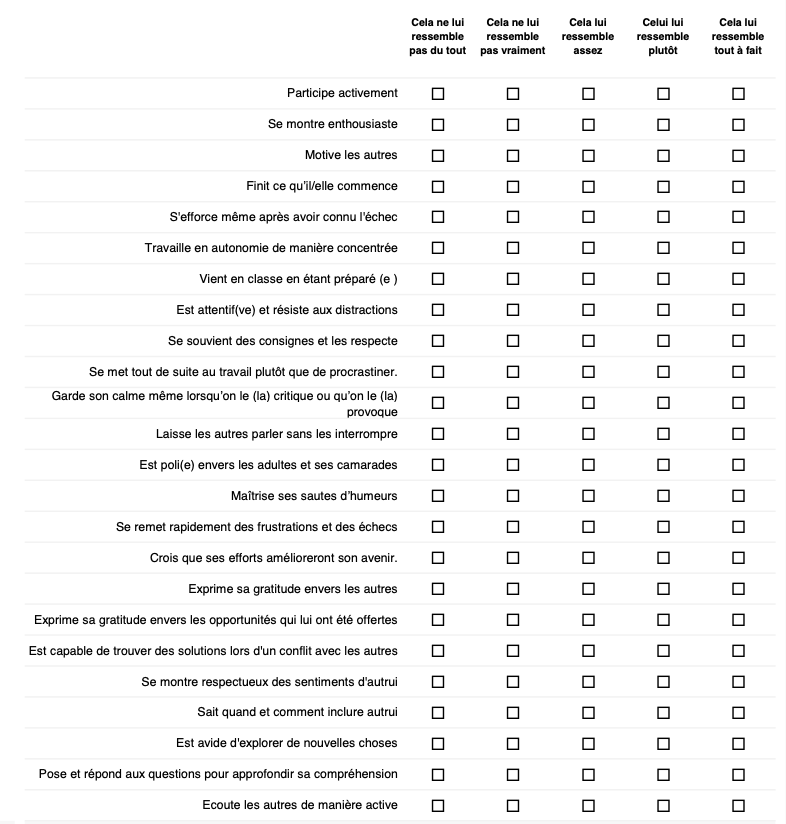


***Figure A3***. Script for research assistant to explain the behavioral task and the student questionnaire to students (both English translation and French version).


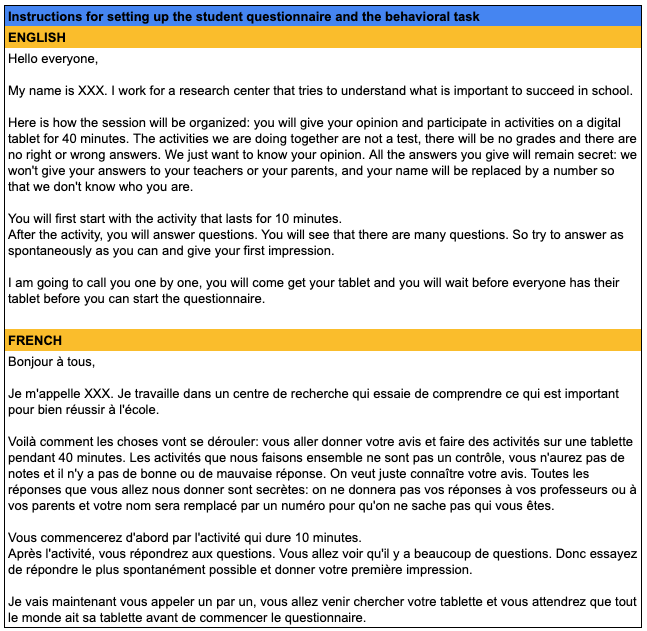


***Figure A4.*** Script for research assistant to explain the behavioral task to students (both English translation and French version)


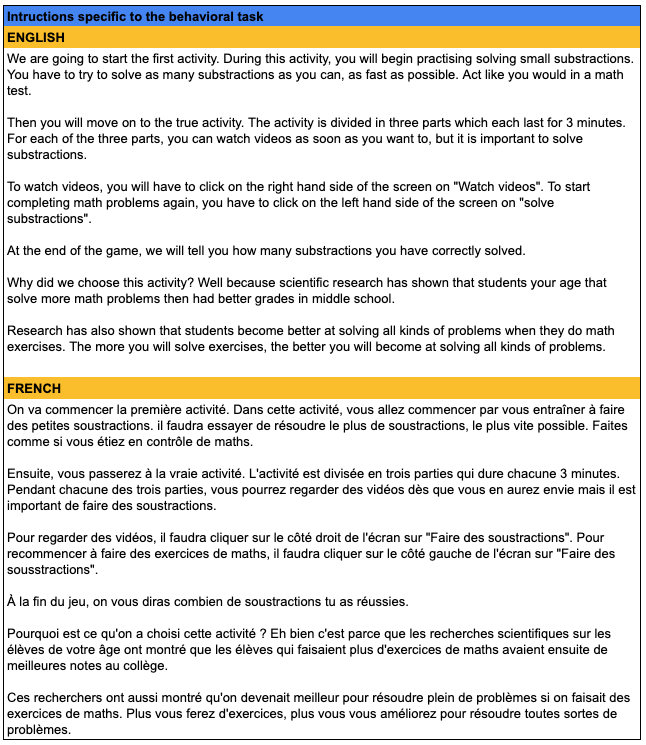


***Figure A5***. Example of screen seen by students solving math problems during the behavioral task. On the right hand side of the screen students could solve simple subtractions. On the left hand side of the screen students could click on "Watch videos"("Regarder des vidéos") at any time.


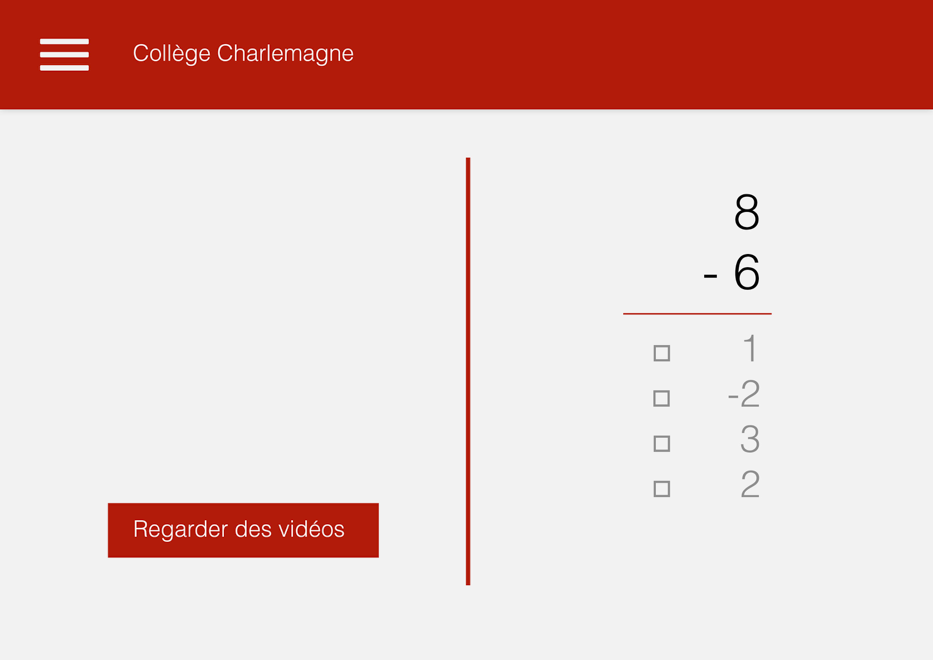


***Figure A6***. Example of screen seen by students watching videos during the behavioral task. On the left hand side of the screen, students could choose the video they wanted to watch. On the right hand side, students could decide to solve math problems by clicking on "Solve subtractions" ("Faire des soustractions").


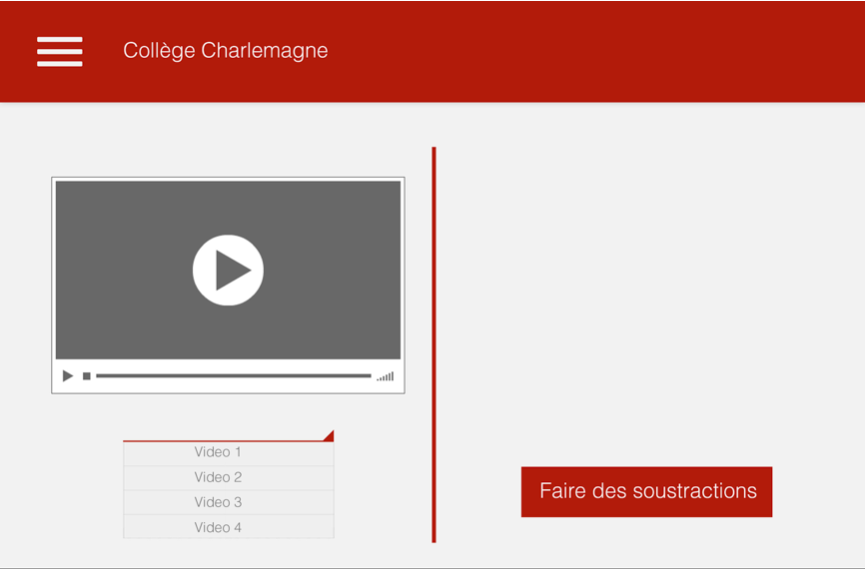


***Figure A7.*** French version of the Domain Specific Impulsivity Scale. The English version can be found in Tsukayama et al. (2013) Tsukayama, Duckworth, and Kim (2013). For student questionnaires, some items in each scale were inverted on the questionnaire to make sure that students were not systematically choosing the same answer. Answers were re-coded and averaged such that a higher score always indicates more agreement with the construct.


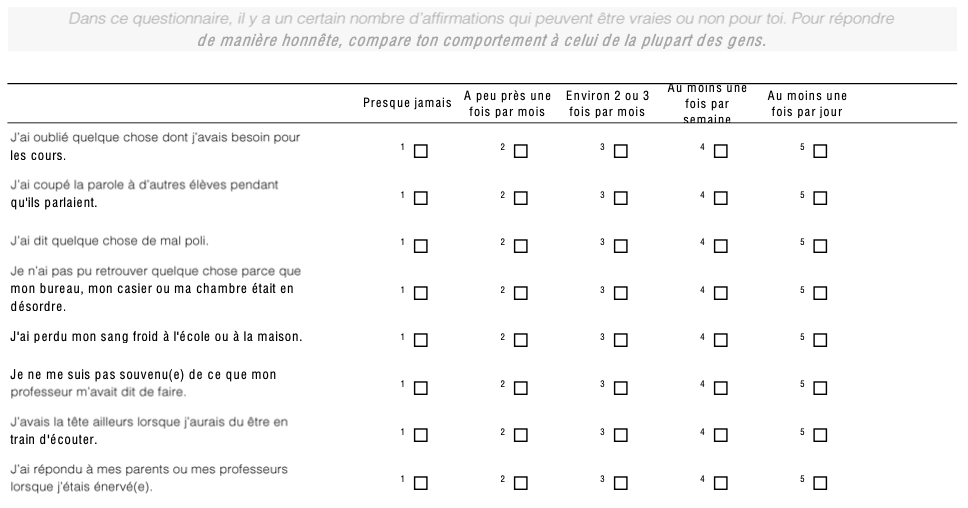


***Figure A8***. French version of the Short Grit Scale. The English version can be found in Duckworth et al. (2009) Duckworth and Quinn (2009). For student questionnaires, some items in each scale were inverted on the questionnaire to make sure that students were not systematically choosing the same answer. Answers were re-coded and averaged such that a higher score always indicates more agreement with the construct.


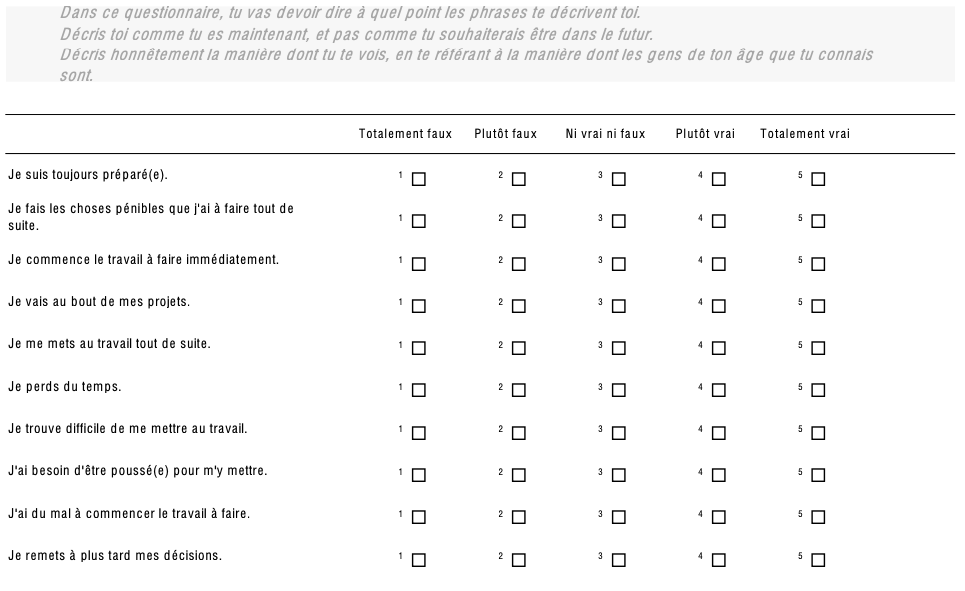


Appendix B

Replication of the Academic Diligence Task

The pre-registration for the replication of the Academic Diligence Task can be found on our OSF page <https://osf.io/afzgx>. Before the beginning of the task, the experimenter explained that solving math problems is important to develop the brain and students were encouraged to solve as many math problems as possible. Students were also told that their answers would be anonymous and confidential, and that they could do whatever they wanted.

***Table B1.*** Descriptive Statistics


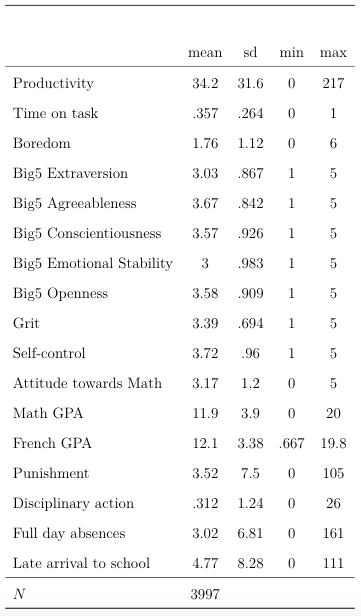


***Table B2.*** Mean values of different variables in each block for the Academic Diligence Task


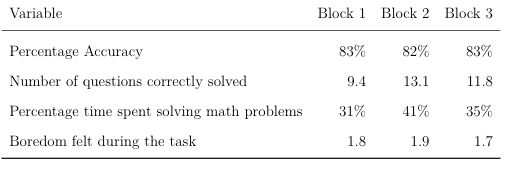


***Table B3.*** Correlation table for performance during each block


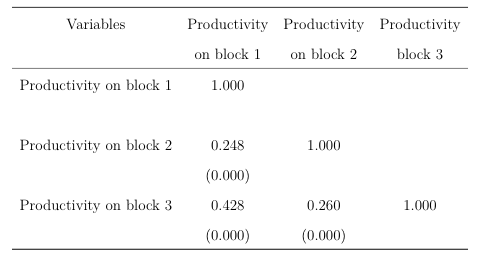


***Table B4.*** Correlation table for time on task during each block


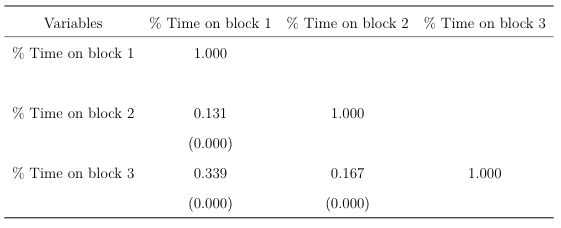


***Table B5.*** Correlation table for boredom during each block


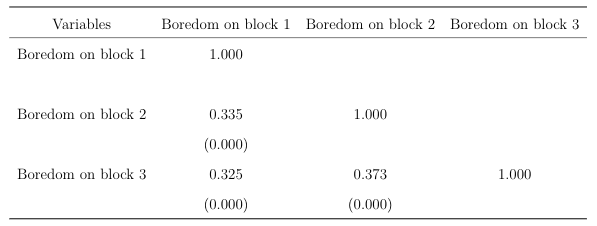


***Table B6.*** Correlation between Performance on ADT measured as the number of questions correctly solved and different traits. *Significantly different from zero at the .10 level,two-tailed test. **Significantly different from zero at the .05 level, two-tailed test.***Significantly different from zero at the .01 level, two-tailed test.


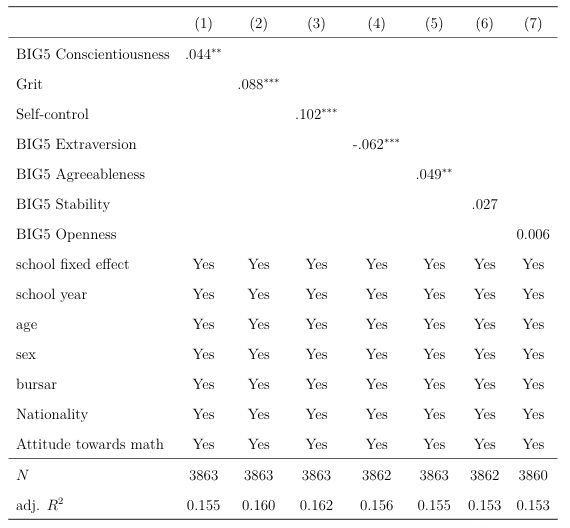


***Table B7.*** Correlation between time spent solving math problems during ADT and different traits.*Significantly different from zero at the .10 level, two-tailed test. **Significantly different from zero at the .05 level, two-tailed test. ***Significantly different from zero at the .01 level, two-tailed test.


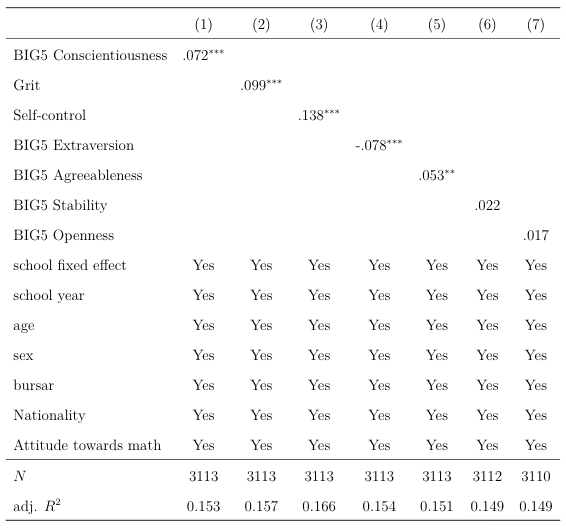


***Table B8.*** Regression of performance on BIG5 agreeableness with self-control and grit as controls.*Significantly different from zero at the .10 level, two-tailed test. **Significantly different from zero at the .05 level, two-tailed test. ***Significantly different from zero at the .01 level, two-tailed test


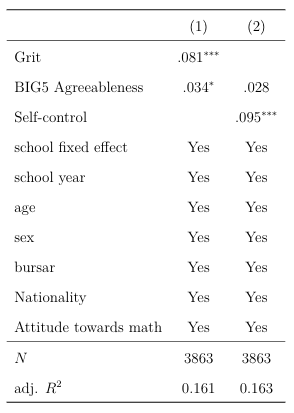


***Table B9.*** Regression of time spent solving math problems during ADT on BIG5 agreeableness with self-control and grit as controls. *Significantly different from zero at the .10 level,two-tailed test. **Significantly different from zero at the .05 level, two-tailed test.***Significantly different from zero at the .01 level, two-tailed test


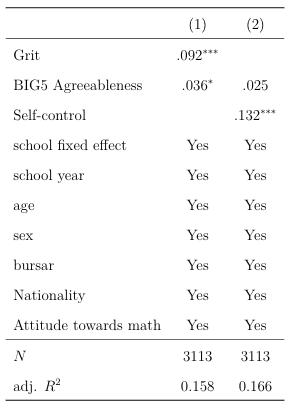


Appendix C

Results

***Table C1.*** Results from expert survey, actual number of votes (and percentage). A link was sent out to researchers via the network of the Paris School of Economics, the École NormaleSupérieure, and Université Paris Dauphine. The survey asked respondents to rank the following three methods according to which would be best to measure non-cognitive skills in middle-schoolers: 1- standardized child self-reported questionnaire, 2- standardized teacher-reported questionnaire, and 3- standardized lab experiment. A total of 114individuals took the survey. 36 of these experts were economists, 45 came from psychology and cognitive sciences and the rest came from other fields such as philosophy of biology. Results did not differ significantly according to the field of the expert.


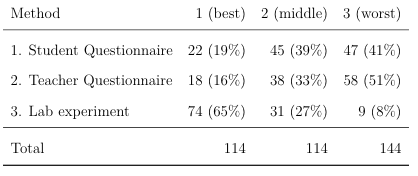


***Figure C1.*** Missing values for the total sample of 6,003 students participating in the study. The data was collected over three years. The first cohort consisted of 784 students in the sixth grade during the spring of 2015. The second cohort consisted of 1,166 students in sixth grade and 1,117 students in seventh grade in the spring of 2016. The third cohort consisted of 930 students in seventh grade in the spring of 2017. Missing values were random, and were mostly due to technical issues downloading data from the school software programs. There were also many missing values for the teacher questionnaire (Character Growth Card) as those were provided in paper format to teachers and then collected by research assistants. There is a high number of missing values for the variable "time on task" for the behavioral task. This is due to a technical problem with the software for students who completed the task in their first year of high school.


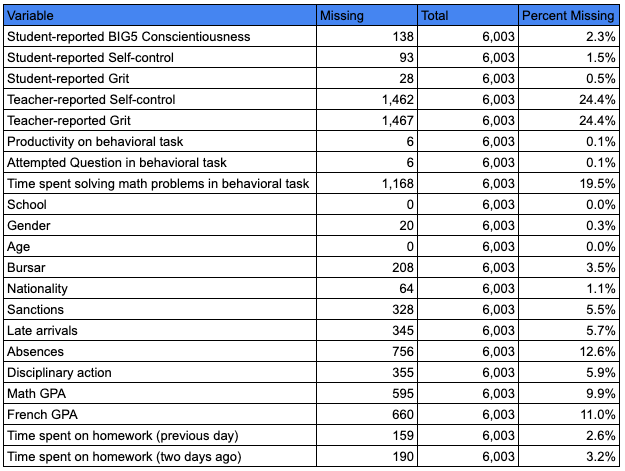


***Table C2.*** Correlation table for different socio-emotional skills, N = 3,997. *Significantly different from zero at the .10 level, two-tailed test. **Significantly different from zero at the .05 level, two-tailed test. ***Significantly different from zero at the .01 level, two-tailed test


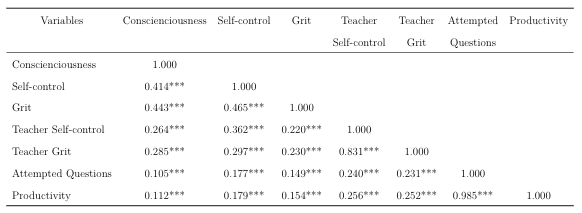


***Table C3.*** Rank-order stability by gender. *Significantly different from zero at the .10 level, two-tailed test. **Significantly different from zero at the .05 level, two-tailed test. ***Significantly different from zero at the .01 level, two-tailed test

| Variables | Female | | Male | |
| --- | --- | --- | --- | --- |
|  | Rank-order stability | N | Rank-order stability | N |
| Conscientiousness | 0.521*** | 729 | 0.456*** | 672 |
| Self-Control | 0.593*** | 735 | 0.526*** | 681 |
| Grit | 0.457*** | 741 | 0.459*** | 692 |
| Teacher Self-control | 0.556*** | 465 | 0.571*** | 440 |
| Teacher Grit | 0.470*** | 465 | 0.510*** | 439 |
| Attempted Questions | 0.453*** | 739 | 0.412*** | 695 |
| Productivity | 0.484*** | 739 | 0.443*** | 695 |
| Time on task | 0.394*** | 458 | 0.341*** | 414 |

***Figure C2.*** Distribution of the different measures of non cognitive skills. First line inthe student-reported measures, second line in the teacher-reported measures and third line is the behavioral task measures.

1. Distribution of student reported conscientiousness score (standardized). N =3,997


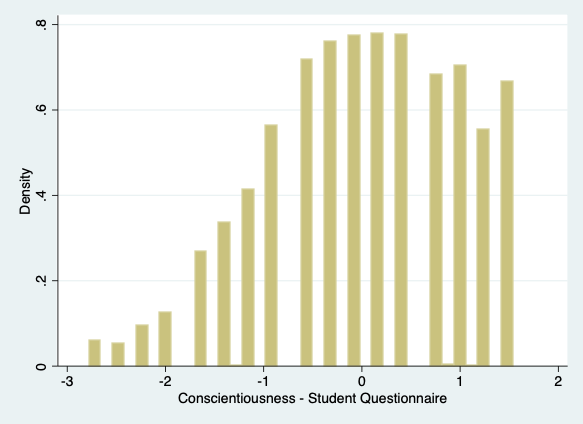


1. Distribution of student reported grit score (standardized). N = 3,997
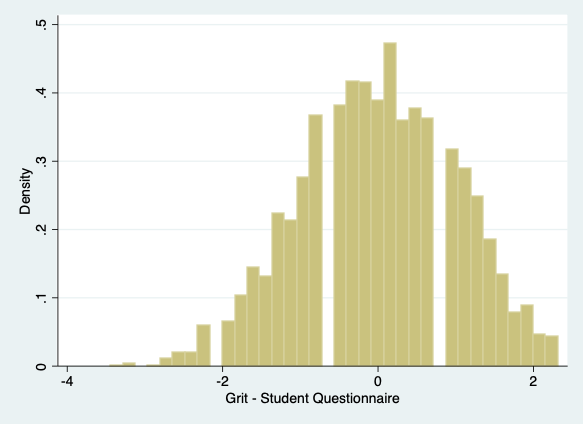

2. Distribution of student reported self-control score (standardized). N = 3,997


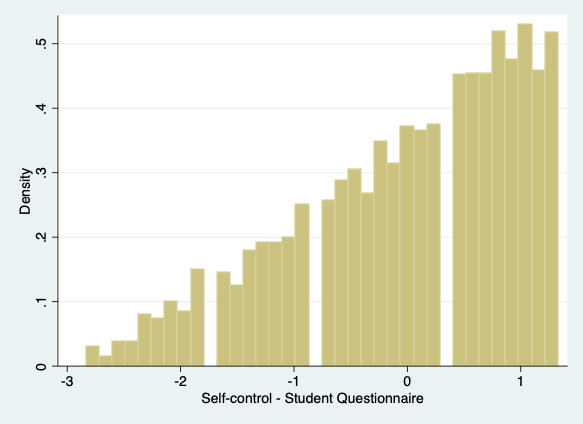


1. Distribution of teacher reported grit score (standardized). N = 3,997


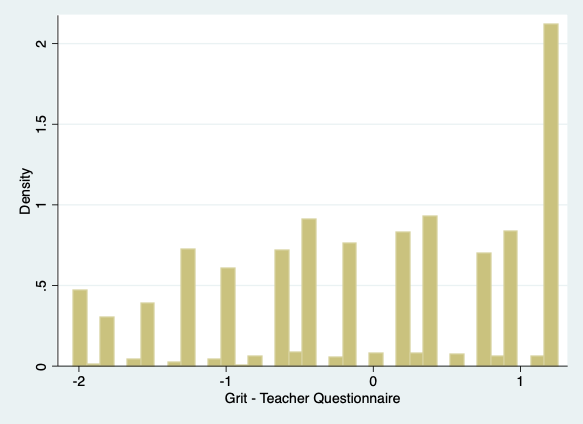


1. Distribution of teacher reported self-control score (standardized). N = 3,997


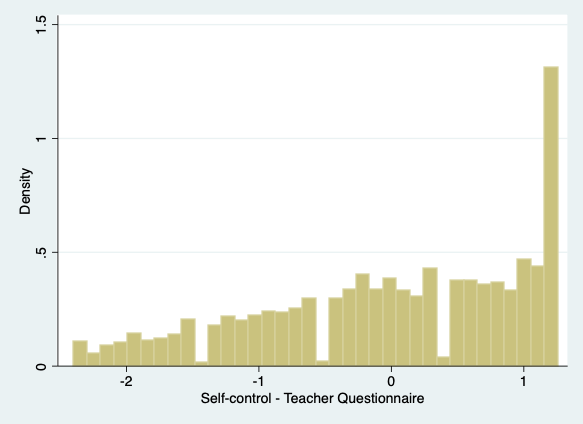


1. Distribution of attempted number of math problem in behavioral task (standardized). N = 3,997


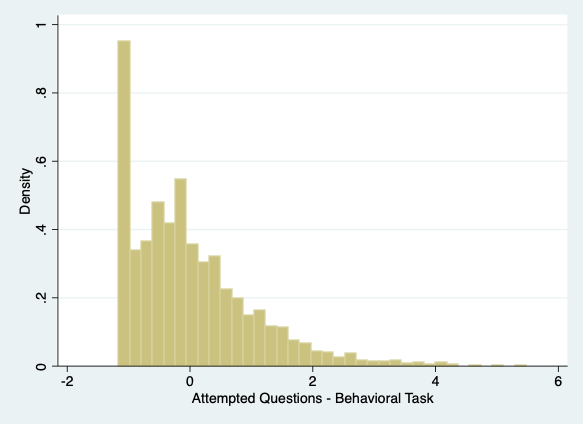


1. Distribution of solved number of math problem in behavioral task (standardized). N = 3,997


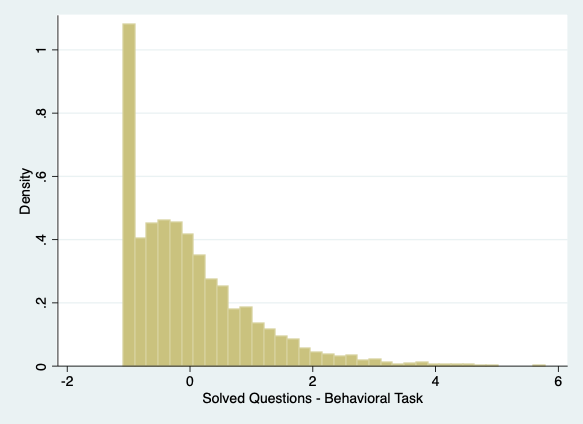


1. Distribution of time spent on solving math problem in behavioral task(standardized). N = 3,213


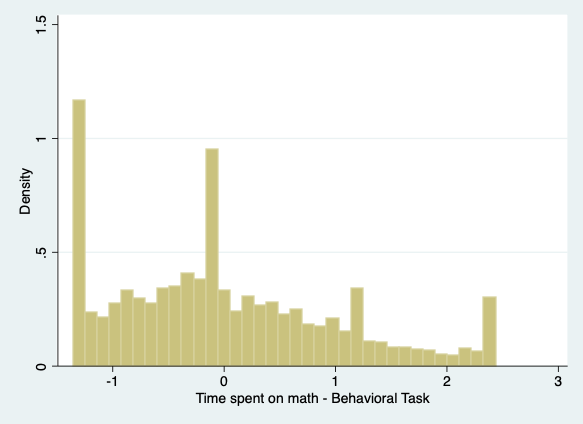


***Figure C3.*** Regression coefficient of standardized disciplinary index on same year socio-emotional skills, no imputations.


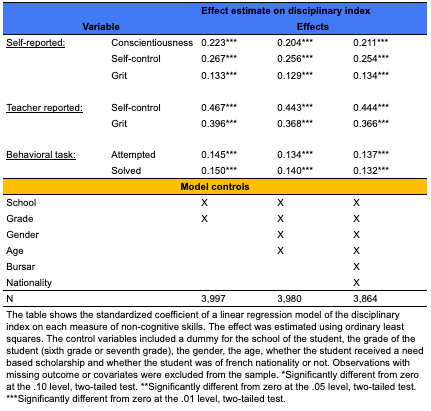


***Figure C4.*** Regression coefficient of time spent doing homework on same year socio-emotional skills, no imputations. Given that time spent doing homework may be sensitive to the day of the week, we recorded the day of the week when the student completed the survey and added it as a control.


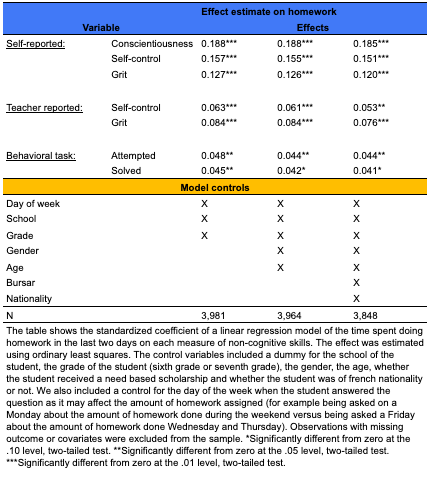


***Figure C5.*** Regression coefficient of math GPA on same year socio-emotional skills, no imputations


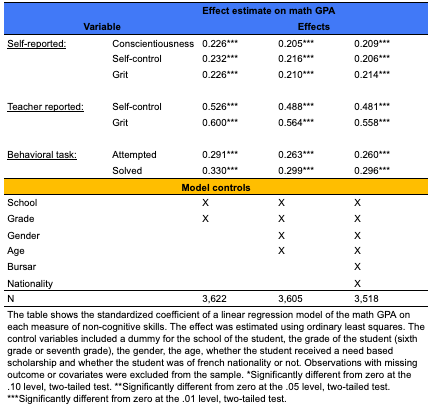


***Figure C6.*** Regression coefficient of French GPA on same year socio-emotional skills, no imputations


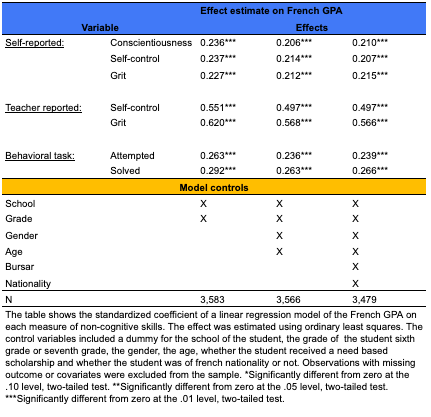


***Figure C7.*** Regression coefficient of standardized disciplinary index on same year socio-emotional skills, with imputations


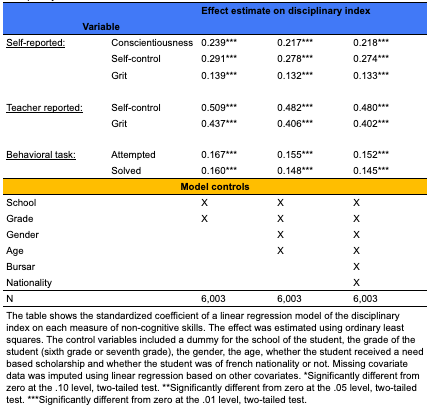


***Figure C8.*** Regression coefficient of time spent doing homework on same year socio-emotional skills, with imputations. Given that time spent doing homework may be sensitive to the day of the week, we recorded the day of the week when the student completed the survey and added it as a control.


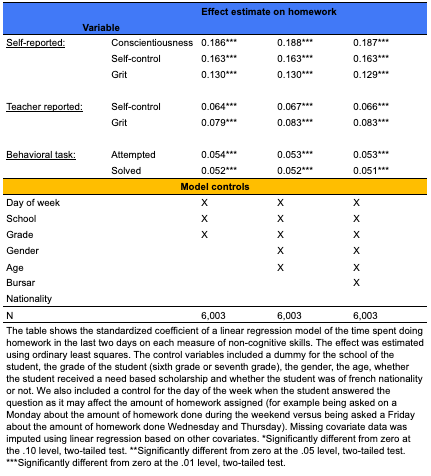


***Figure C9.*** Regression coefficient of math GPA on same year socio-emotional skills, with imputations.


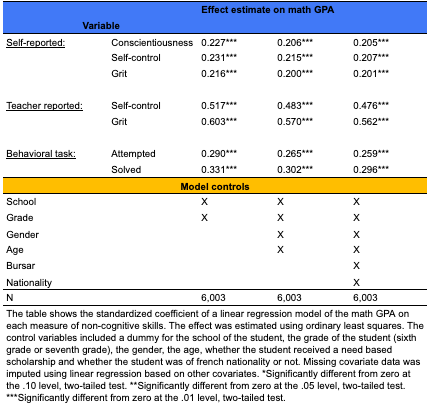


***Figure C10.*** Regression coefficient of French GPA on same year socio-emotional skills, with imputations


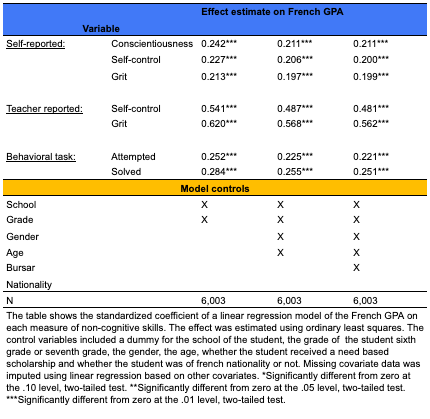


***Figure C11.*** Regression coefficient of standardized disciplinary index on previous year socio-emotional skills, controlling for previous year standardized disciplinary index.


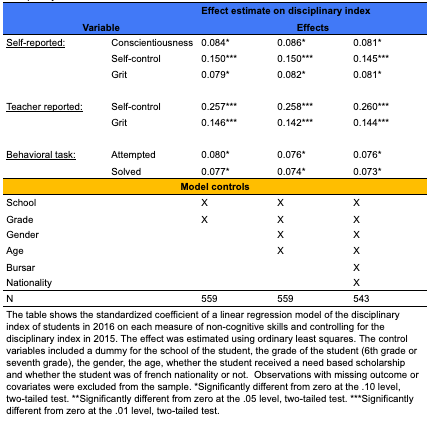


***Figure C12.*** Regression coefficient of time spent doing homework on previous year socio-emotional skills, controlling for previous year time spent doing homework. Given that time spent doing homework may be sensitive to the day of the week, we recorded the day of the week when the student completed the survey and added it as a control.


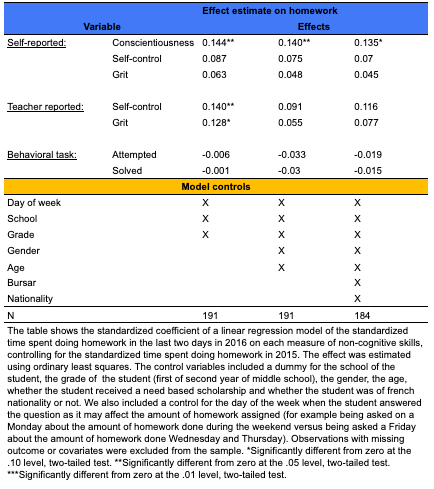


***Figure C13.*** Regression coefficient of math GPA on previous year socio-emotional skills, controlling for previous year math GPA.


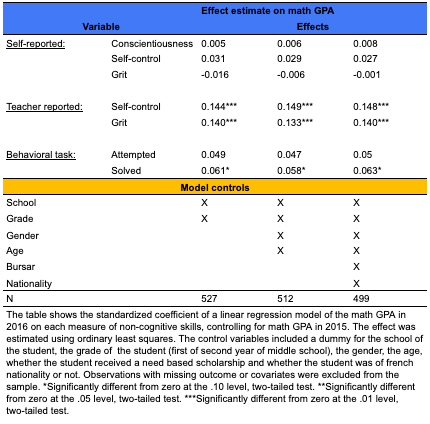


***Figure C14.*** Regression coefficient of French GPA on previous year socio-emotional skills, controlling for previous year French GPA.


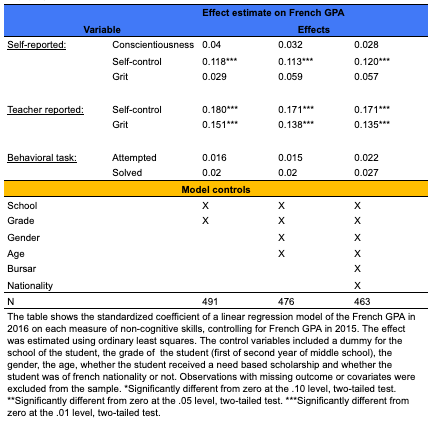


***Figure C15.*** Regression coefficient of standardized disciplinary index on socio-emotional skills measured in year X, controlling for year X standardized disciplinary index.


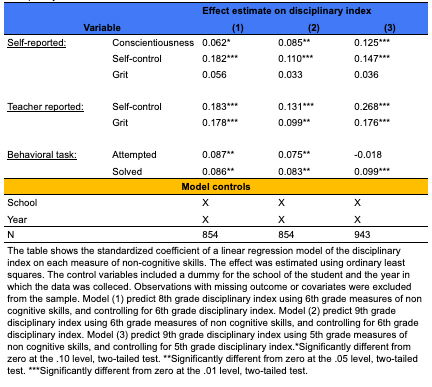


***Figure C16.*** Regression coefficient of time spent doing homework on socio-emotional skills measured in year X, controlling for year X time spent doing homework. Given that time spent doing homework may be sensitive to the day of the week, we recorded the day of the week when the student completed the survey and added it as a control.


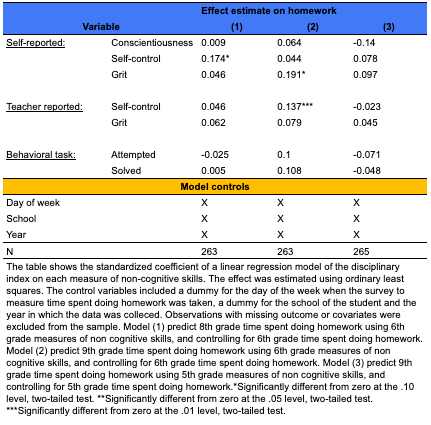


***Figure C17***. Regression coefficient of math GPA on socio-emotional skills measured in year X, controlling for year X math GPA.


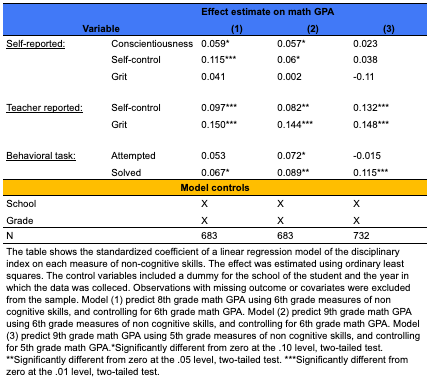


***Figure C18.*** Regression coefficient of French GPA on socio-emotional skills measured in year X, controlling for year X French GPA.


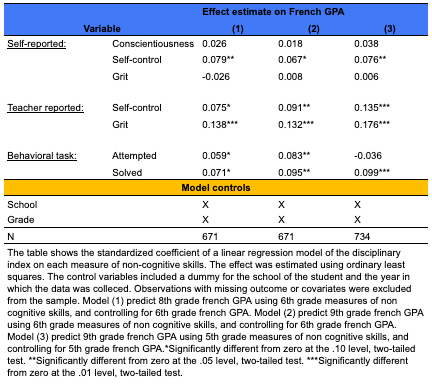


References

Duckworth, A. L., & Quinn, P. D. (2009, February). Development and Validation of the Short Grit Scale (Grit–S). *Journal of Personality Assessment*, 91(2), 166–174. Retrieved 2020-03-04, from<http://www.tandfonline.com/doi/abs/10.1080/00223890802634290doi:10.1080/00223890802634290>

Park, D., Tsukayama, E., Goodwin, G. P., Patrick, S., & Duckworth, A. L. (2017,January). A tripartite taxonomy of character: Evidence for intrapersonal, interpersonal, and intellectual competencies in children. *Contemporary Educational Psychology*, 48, 16–27. Retrieved 2020-03-03, from <https://linkinghub.elsevier.com/retrieve/pii/S0361476X16300352doi:10.1016/j.cedpsych.2016.08.001>

Tsukayama, E., Duckworth, A. L., & Kim, B. (2013, May). Domain-specific impulsivity in school-age children. *Developmental Science*,16(6), 879–893. Retrieved 2020-03-04, from <http://doi.wiley.com/10.1111/desc.12067doi:10.1111/desc.12067>
